# Supplementary material for: Epigenetic profiling reveals key genes and cis-regulatory networks specific to human parathyroids
Source: Nat Commun. 2024 Mar 7;15:2106. doi: 10.1038/s41467-024-46181-3 (PMC10920874; doi:10.1038/s41467-024-46181-3)
Supplement: Supplementary file 1 — Supplementary Information [file 41467_2024_46181_MOESM1_ESM.pdf]

Epigenetic Profiling Reveals Key Genes and Cis-Regulatory Networks Specific to Human Parathyroids

Youngsook L Jung, Wenping Zhao, Ian Li, Dhawal Jain, Charles B Epstein, Bradley E Bernstein, Sareh Parangi, Richard Sherwood, Cassianne Robinson-Cohen, Yi-Hsiang Hsu, Peter J Park, Michael Mannstadt

Supplementary Figures

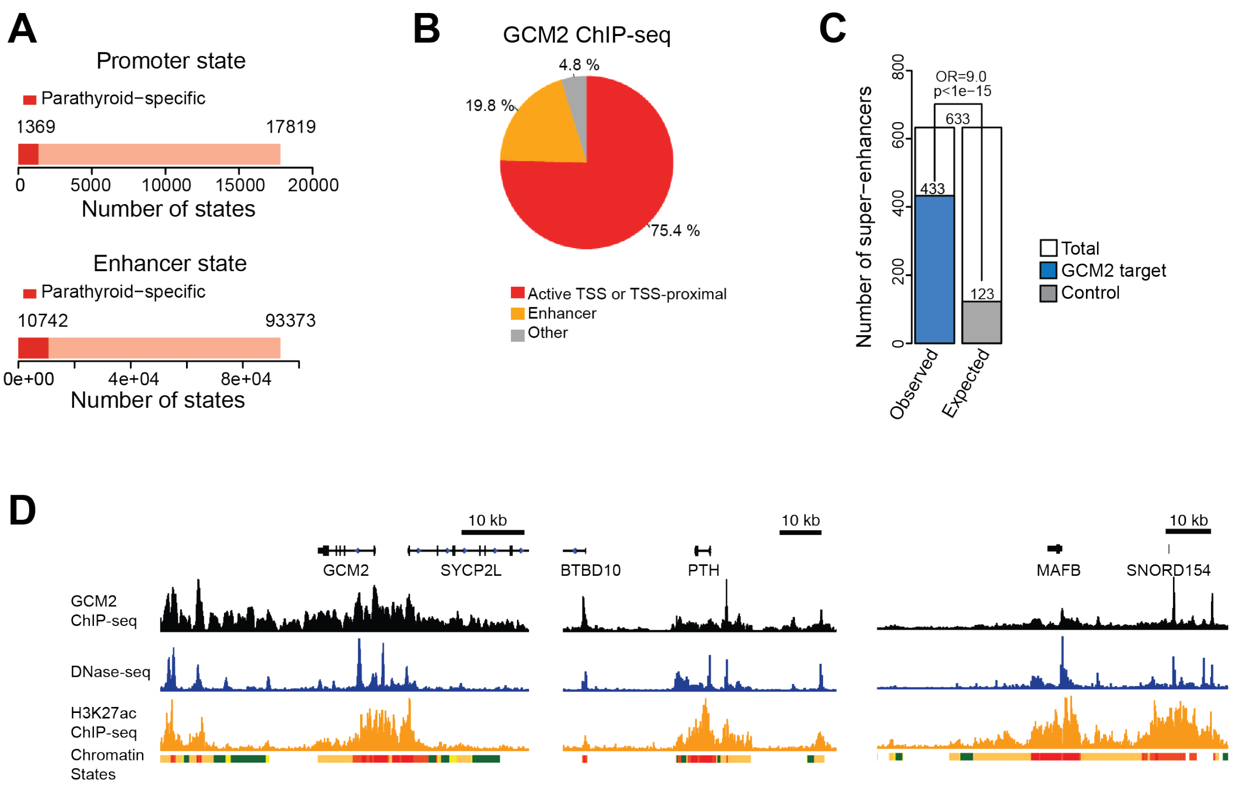

Supplementary Figure 1. The landscape of chromatin states and GCM2 binding in human parathyroids. Related to Figure 1. A, Number of chromatin states of promoters and enhancers in parathyroids. Red, parathyroid-specific elements compared to 98 other cell/tissues profiled in the Roadmap Epigenomics project. **B,** Proportions of chromatin states associated with GCM2 binding. **C,** Number of super-enhancers targeted by GCM2 compared to random expectation (p-value by a one-sided Fisher's

Exact test). **D,** Profiles of ChIP-seq of GCM2 binding (black), DNase-seq (blue), and H3K27ac (orange), and chromatin states for the genes of GCM2 (left), PTH (middle) and MAFB (right) in parathyroids. These genes are previously known core genes in parathyroids. Strong and broad (>10kb) H3K27ac regions indicate these genes contain super-enhancers. Y-axis: normalized read densities for *GCM2* ChIP-seq and DNA-seq profiles.

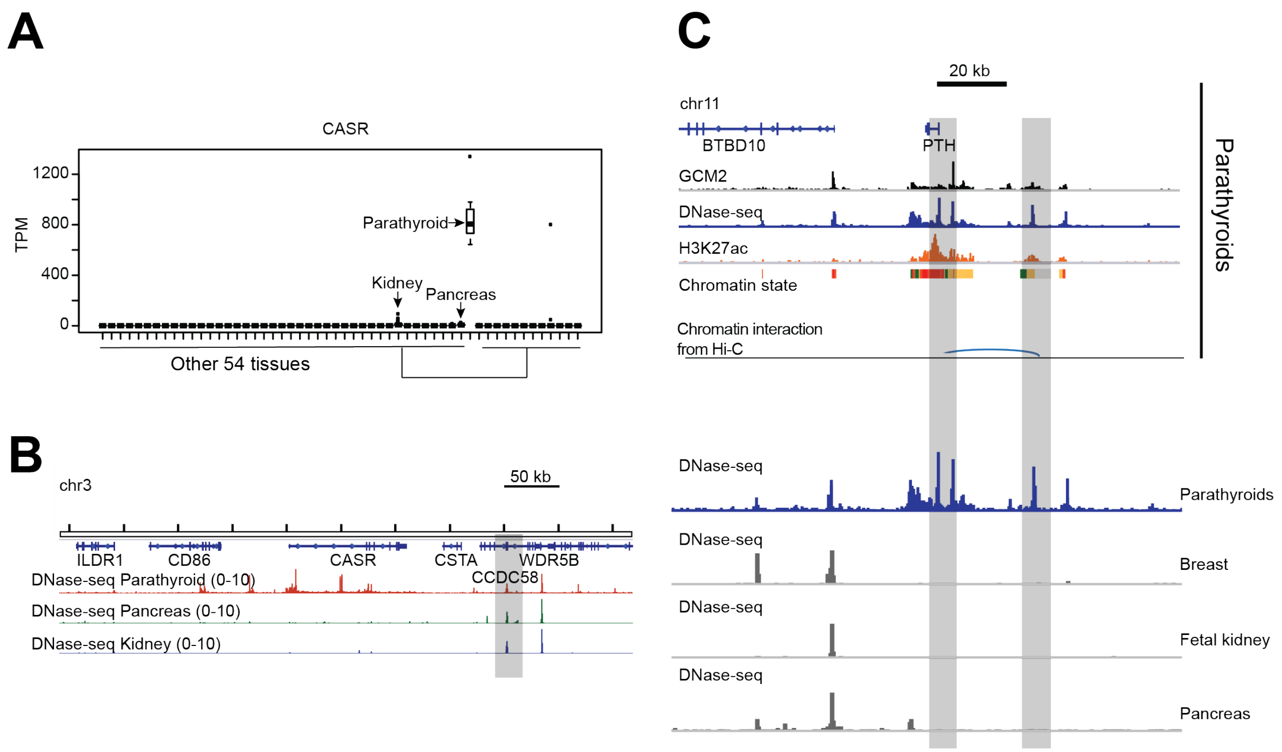

Supplementary Figure 2. Examples of parathyroid-specific functional elements and gene expression. Related to Figure 2. A, Expression levels of CASR gene across 55 tissues. This gene is the most highly transcribed in parathyroids but is expressed in several tissues including kidney and pancreas. n = 8, biologically independent samples for parathyroids. The box represents the interquartile range (IQR) divided by the median, and Tukey whiskers extend to a maximum of 1.5 × IQR beyond the box. **B,** Open chromatin profiles around CASR in tissues which express CASR**.** Y-axis: normalized read densities of DNase-seq. The scales of the values are in parenthesis. The signals were scaled for the comparison between tissues. A peak at the

promoter of CCDC58 that is universally expressed with similar expression levels across tissues serves as a reference peak (highlighted). **C**, Chromatin environment around PTH showing GCM2, DNase-seq, H3K27ac, chromatin states and Hi-C chromatin interaction in parathyroids. Blow tracks show DNase-seq from other tissue types such as breast, fetal kidney and pancreas. There is a distal enhancer region of PTH that interacts with the promoter of PTH. This distal enhancer element is parathyroid-specific. Gray boxes highlight the promoter of PTH and its distal enhancer in parathyroids.

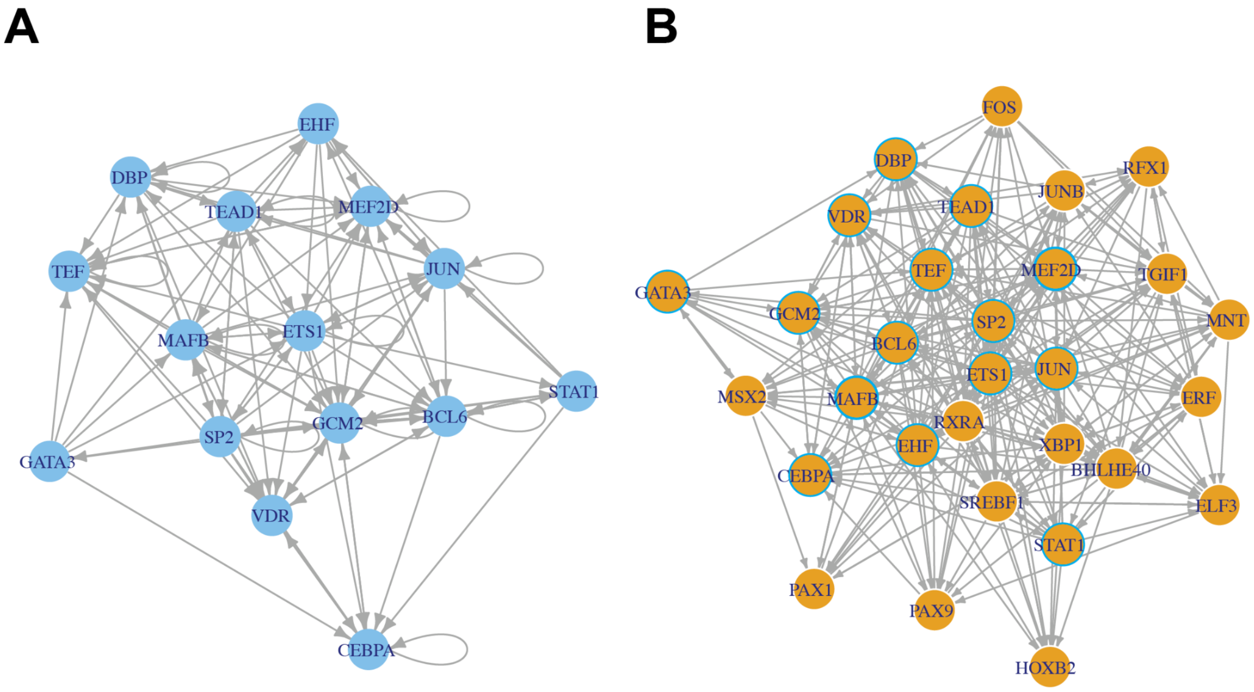

Supplementary Figure 3. Core cis-regulation circuit in parathyroids. Related to Figure 3. A, Core TF regulation circuit in parathyroids centered on GCM2. A different representation of Figure 3E. These TFs were predicted from the motif sequences of GCM2 regulatory elements. TFs having more connections are located closer. **B**, Extended core TF regulation circuit in parathyroids. This circuit included targets co-regulated by GCM2 and other TFs. Blue circles indicate overlapping TFs with those in Figure S3A. Note that PAX1 and PAX9 are targets of GCM2 and its co-regulators.

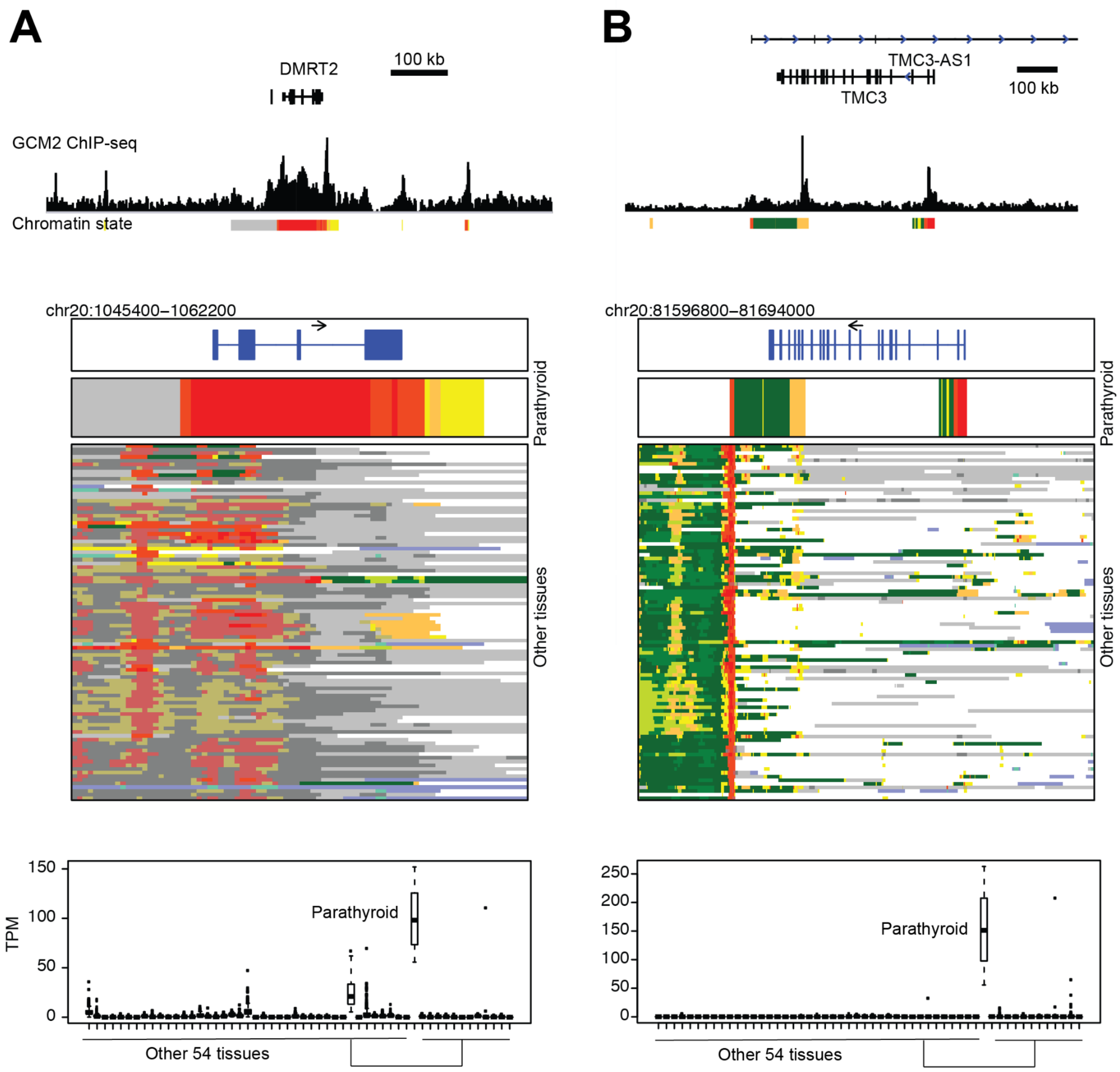

Supplementary Figure 4. Identification of genes previously not known to play a role in the parathyroids. Related to Figure 4. A, GCM2 binding and chromatin states (top) for *DMRT2*. The arrow indicates the transcription starting site and transcriptional direction. Chromatin states for the *DMRT2* locus in the parathyroid and other tissues from the Roadmap Epigenomics (middle). Expression levels in the parathyroid and

other tissues from the GTEx project (bottom). Orange: parathyroid, Gray: other tissue types from the GTEx project. n = 8, biologically independent samples for parathyroids. The box represents the interquartile range (IQR) divided by the median, and Tukey whiskers extend to a maximum of 1.5 × IQR beyond the box. The colors in the middle panel indicate chromatin states as in Figure 4B. Although *DMRT2* is expressed in many tissues, this gene has a higher enhancer activity (exclusive super-enhancer) and a higher transcriptional activity in parathyroids. **B**, Same as in A but for *TMC3*. This gene is specifically expressed in parathyroids.

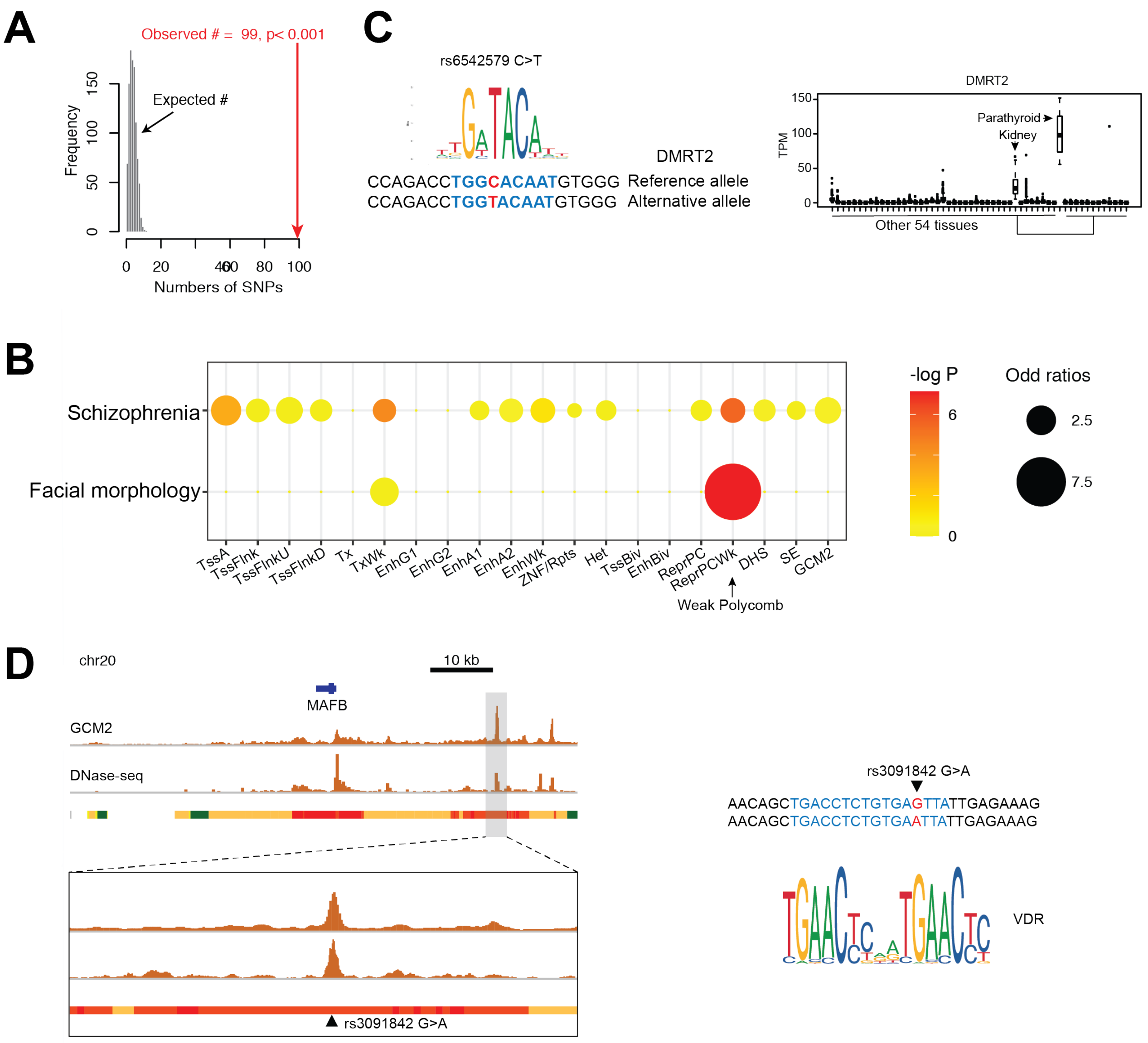

Supplementary Figure 5. GWAS SNPs associated with serum PTH concentrations that are located in parathyroid-specific regulatory elements. Related to Figure 5. **A**, PTH level-associated SNPs in super-enhancers in parathyroids. The histogram of expected numbers of PTH SNPs by chance. The red vertical line indicates the observed number of PTH SNPs. A *p*-value from a one-sided permutation test. **B,** GWAS SNPs associated with facial morphology and schizophrenia, traits that are not related to parathyroid function, in regulatory elements of parathyroids (negative control). *P*-values by a one-sided Fisher-exact test. The sizes of circles correspond to odds ratios and colors to *p*-values. **C**, Left: Motif sequence of *DMRT2* around rs6542579, for which the binding affinity change by the SNP was predicted. Right: Expression levels of *DMRT2* compared to other tissues. n = 8, biologically independent samples for parathyroids. The box represents the interquartile range (IQR) divided by the median, and Tukey whiskers extend to a maximum of 1.5 × IQR beyond the box. The expression levels of *DMRT2* were highest in parathyroids while this gene is also expressed in several tissues including kidneys. **D**, PTH level-associated SNP (rs3091842) in the parathyroid-specific intronic enhancer of *MAFB*. Profiles of GCM2 ChIP-seq, DNase-seq, and chromatin states. Left: The PTH-level SNP overlaps the GCM2 peak and parathyroid-specific DHS. Right: the motif sequence of *VDR* around rs3091842, for which the binding affinity change by the SNP was predicted.
