# Supplementary material for: Epigenetic profiling reveals key genes and cis-regulatory networks specific to human parathyroids
Source: Nat Commun. 2024 Mar 7;15:2106. doi: 10.1038/s41467-024-46181-3 (PMC10920874; doi:10.1038/s41467-024-46181-3)
Supplement: Supplementary file 3 — Description of Additional Supplementary Files [file 41467_2024_46181_MOESM3_ESM.pdf]

Description of Additional Supplementary Files

File Name: Supplementary Data 1
Description: GCM2 ChIP-seq peaks from merged replicates in human parathyroid glands, related to Figure 1.

File Name: Supplementary Data 2
Description: Super-enhancers in parathyroid glands defined by the high signal intensities of H3K27ac, related to Figure 1.

File Name: Supplementary Data 3
Description: Enriched biological process terms enriched for super-enhancers in parathyroid glands, related to Figure 1. P-values by a two-sided binomial test.

File Name: Supplementary Data 4
Description: Genes associated with super-enhancers that are highly specific to parathyroids (present in no more than one other tissue type compared to 98 other cell/tissue types), related to Figure 1.

File Name: Supplementary Data 5
Description: Significant chromatin interactions with FDR=0.1 from Hi-C data in parathyroids, related to Figure 2.

File Name: Supplementary Data 6
Description: Primer information used in enhancer validation experiments in vitro. Putative enhancers were identified utilizing H3K27ac and GCM2 peaks, related to Figure 2.

File Name: Supplementary Data 7
Description: Significantly enriched TF motifs identified in open chromatin in parathyroids. Only TFs expressed in parathyroids (TPM>50) were selected. P-values by a two-sided Rank sum test and adjusted p-values are provided.

File Name: Supplementary Data 8
Description: Genes with transcriptional specificity in the parathyroids. We compared the expression levels of genes in the parathyroids with those in 54 other tissues. We selected genes with z-scores > 7 across tissues and TPM > 50 in parathyroids, related to Figure 4.

File Name: Supplementary Data 9
Description: GWAS significance levels and references for SNPs examined in this study, related to Figure 5. P-values by a two-sided Hardy–Weinberg equilibrium test.

File Name: Supplementary Data 10
Description: Information on the antibodies used for ChIP-seq experiments in this study.
