# Supplementary material for: Epigenetic profiling reveals key genes and cis-regulatory networks specific to human parathyroids
Source: Nat Commun. 2024 Mar 7;15:2106. doi: 10.1038/s41467-024-46181-3 (PMC10920874; doi:10.1038/s41467-024-46181-3)
Supplement: Supplementary file 5 — Reporting Summary [file 41467_2024_46181_MOESM5_ESM.pdf]

nature portfolio

Corresponding author(s): Youngsook Lucy Jung, Michael Mannstadt

Last updated by author(s): Jan 30, 2024

Reporting Summary

Nature Portfolio wishes to improve the reproducibility of the work that we publish. This form provides structure for consistency and transparency in reporting. For further information on Nature Portfolio policies, see our Editorial Policies and the Editorial Policy Checklist.

Statistics

For all statistical analyses, confirm that the following items are present in the figure legend, table legend, main text, or Methods section.

n/a	Confirmed	
☐	☒	The exact sample size (*n*) for each experimental group/condition, given as a discrete number and unit of measurement
☐	☒	A statement on whether measurements were taken from distinct samples or whether the same sample was measured repeatedly
☐	☒	The statistical test(s) used AND whether they are one- or two-sided<br>*Only common tests should be described solely by name; describe more complex techniques in the Methods section.*
☒	☐	A description of all covariates tested
☐	☒	A description of any assumptions or corrections, such as tests of normality and adjustment for multiple comparisons
☐	☒	A full description of the statistical parameters including central tendency (e.g. means) or other basic estimates (e.g. regression coefficient) AND variation (e.g. standard deviation) or associated estimates of uncertainty (e.g. confidence intervals)
☐	☒	For null hypothesis testing, the test statistic (e.g. *F*, *t*, *r*) with confidence intervals, effect sizes, degrees of freedom and *P* value noted<br>*Give P values as exact values whenever suitable.*
☒	☐	For Bayesian analysis, information on the choice of priors and Markov chain Monte Carlo settings
☒	☐	For hierarchical and complex designs, identification of the appropriate level for tests and full reporting of outcomes
☒	☐	Estimates of effect sizes (e.g. Cohen's *d*, Pearson's *r*), indicating how they were calculated

Our web collection on statistics for biologists contains articles on many of the points above.

Software and code

Policy information about availability of computer code

Data collection	No software used for data collection
Data analysis	Bowtie ver.1.1.2 for read alignment, Hotspot for DNase-seq peak detection, MACS for ChIP-seq peak detection, HICCUP for Hi-C data process were used. R codes (ver.4.2.1) for downstream analysis are deposited in github (https://github.com/YLucyJung/PTG).

For manuscripts utilizing custom algorithms or software that are central to the research but not yet described in published literature, software must be made available to editors and reviewers. We strongly encourage code deposition in a community repository (e.g. GitHub). See the Nature Portfolio guidelines for submitting code & software for further information.

Data

Policy information about availability of data

All manuscripts must include a data availability statement. This statement should provide the following information, where applicable:

- Accession codes, unique identifiers, or web links for publicly available datasets
- A description of any restrictions on data availability
- For clinical datasets or third party data, please ensure that the statement adheres to our policy

This study was registered, and the sequencing data were submitted to dbGaP (https://www.ncbi.nlm.nih.gov/projects/gap/cgi-bin/study.cgi?study_id=phs003302.v1.p1). Authorized requests are required for data access following the dbGaP request form.

Research involving human participants, their data, or biological material

Policy information about studies with human participants or human data. See also policy information about sex, gender (identity/presentation), and sexual orientation and race, ethnicity and racism.

Reporting on sex and gender	Tissue was collected with an assigned code number, and although the gender of the donor is unknown, no gender was specifically excluded
Reporting on race, ethnicity, or other socially relevant groupings	Tissue was collected with an assigned code number, and although the ethnicity of the donor is unknown, the majority of patients seen at this institution are of Caucasian ethnicity and most patients undergoing parathyroidectomy are over 60 years old
Population characteristics	Tissues were collected from adult individuals with a parathyroid adenoma.
Recruitment	The study included individuals diagnosed with hyperparathyroidism who were undergoing surgical removal of a parathyroid adenoma (parathyroidectomy).
Ethics oversight	Human parathyroid adenoma tissues were collected with informed consent at Massachusetts General Hospital under protocol 2008P001466.

Note that full information on the approval of the study protocol must also be provided in the manuscript.

Field-specific reporting

Please select the one below that is the best fit for your research. If you are not sure, read the appropriate sections before making your selection.

☒ Life sciences ☐ Behavioural & social sciences ☐ Ecological, evolutionary & environmental sciences

For a reference copy of the document with all sections, see nature.com/documents/nr-reporting-summary-flat.pdf

Life sciences study design

All studies must disclose on these points even when the disclosure is negative.

Sample size	We used a total of 20 samples, with 2-8 samples for each method, a typical approach in human epigenomic studies, such as those conducted in projects like ENCODE.
Data exclusions	We excluded samples with low quality control (QC) results from the analysis, such as those exhibiting low signal-to-noise ratios or inconsistent peaks when compared to other replicates.
Replication	Depending on the methods, we used 2-8 biological replications. One ChIP-seq and two ATAC-seq data were discarded due to low QC.
Randomization	We did not employ randomization for data collection or group allocation, as we used all data collected due sample sparsity.
Blinding	Investors were blinded during data collection or group allocation.

Reporting for specific materials, systems and methods

We require information from authors about some types of materials, experimental systems and methods used in many studies. Here, indicate whether each material, system or method listed is relevant to your study. If you are not sure if a list item applies to your research, read the appropriate section before selecting a response.

Materials & experimental systems

n/a	Involved in the study	
☐	☒	Antibodies
☐	☒	Eukaryotic cell lines
☒	☐	Palaeontology and archaeology
☒	☐	Animals and other organisms
☒	☐	Clinical data
☒	☐	Dual use research of concern
☒	☐	Plants

Methods

n/a	Involved in the study	
☐	☒	ChIP-seq
☒	☐	Flow cytometry
☒	☐	MRI-based neuroimaging

Antibodies

Antibodies used	We used the GCM2 (S-19) sc-79496 antibody (Santa Cruz) or normal goat IgG sc-2028 IgG (Santa Cruz) for the GCM2 ChIP-seq experiments.
Validation	Detailed information regarding antibody validation can be found in the following sources: https://datasheets.scbt.com/sc-79496.pdf Additionally, we conducted computational validation through GCM2 motif analysis.

Eukaryotic cell lines

Policy information about cell lines and Sex and Gender in Research

Cell line source(s)	HEK293 (ATCC) and DF-1 (ATCC)
Authentication	None of the cell lines were authenticated. Cell lines were negative for mycoplasma.
Mycoplasma contamination	
Commonly misidentified lines (See ICLAC register)	No commonly misidentified cell lines were used in the study.

Plants

Seed stocks	NA
Novel plant genotypes	NA
Authentication	NA

ChIP-seq

Data deposition

☒ Confirm that both raw and final processed data have been deposited in a public database such as GEO.

☒ Confirm that you have deposited or provided access to graph files (e.g. BED files) for the called peaks.

Data access links *May remain private before publication.*	https://www.ncbi.nlm.nih.gov/projects/gap/cgi-bin/study.cgi?study_id=phs003302.v1.p1
Files in database submission	fastq or bam files for 2 ChIP-seq, 3 Dnase-seq, 5 ATAC-seq, and 8 RNA-seq datasets.
Genome browser session (e.g. UCSC)	NA

Methodology

Replicates	We used two biological replicates from two different individuals for the analysis.
Sequencing depth	The total read depth from two biological replicates was 175M with paired-end reads, out of which 141M reads were uniquely mapped. The read length was 50 bp.
Antibodies	We used the GCM2 (S-19) sc-79496 antibody (Santa Cruz) or normal goat IgG sc-2028 IgG (Santa Cruz) for the GCM2 ChIP-seq experiments.
Peak calling parameters	We aligned the reads to the hg19 genome using Bowtie with unique mapping options. For subsequent analyses, we exclusively used uniquely mappable reads. Significant peaks were detected using MACS2 callpeak with a q-value threshold of 0.01.
Data quality	We ensured data quality by assessing the consistency between replicates and conducting the motif analysis. Total 10,112 singnificant peaks were detected at fold enrichment > 5 and FDR < 0.05.
Software	We used Bowtie version 1.2.2 for read alignment, MACS2 version 1.4 for peak calling, GREAT for gene ontology analysis, and MEME for motif analysis. Customized analysis was done using R codes, which have been deposited at https://github.com/YLucyJung/PTG.
